# Literacy and recent history of diarrhoea are predictive of *Plasmodium falciparum *parasitaemia in Kenyan adults

**DOI:** 10.1186/1475-2875-5-96

**Published:** 2006-11-01

**Authors:** Rodney L Coldren, Trish Prosser, Fredrick Ogolla, Victor O Ofula, Nicholas Adungo

**Affiliations:** 1 United States Army Medical Research Unit – Kenya, Mbagathi Rd, Nairobi, Kenya; 2 Edith Cowan University, 100 Joondalup Drive, Joondalup, WA, Australia; 3 Centre for Infectious and Parasitic Diseases Control Research, Kenya Medical Research Institute, Busia, Kenya; 4 Centre for Clinical Research, Kenya Medical Research Institute, Mbagathi Rd, Nairobi, Kenya

## Abstract

**Background:**

Malaria is one of the most serious health problems in Kenya. In 2004, the Kenya Medical Research Institute and the US Army Medical Research Unit – Kenya surveyed adults in Samburu, Malindi, and Busia districts to determine socioeconomic risk factors for infection.

**Methods:**

Sociodemographic, health, and antimalarial data were collected along with blood for malaria testing. A smear was considered negative only if no *Plasmodium falciparum *parasites were observed in 100 high-powered fields. Univariate analysis was performed with Pearson's Chi-square test and univariate logistic regression. A multivariate logistic regression model was then created which included only variables found to be at least marginally significant in univariate analysis.

**Results:**

A total of 1,141 subjects were recruited: 238 from Samburu, 442 from Malindi, and 461 from Busia. Smear positivities for *P. falciparum *were 1.7% in Samburu, 7.2% in Malindi and 22.3% in Busia. Interdistrict differences were statistically significant (p < 0.001) in univariate analysis and in a multivariate logistic regression model which included district, literacy, occupation, and recent illness as independent variables. In the model, literacy and recent diarrhoeal illness were positively and at least marginally significantly associated with parasitaemia (p = 0.023 and p = 0.067, respectively). Neither age, sex, occupation, history of malaria in the previous three months, nor use of antimalarials in the previous four weeks were significantly associated with parasitaemia.

**Conclusion:**

While district of residence was the variable most highly predictive for parasitaemia among Kenyan adults surveyed, both a recent history of diarrhoeal illness and literacy were at least marginally statistically significant predictors.

## Background

Malaria is one of the most serious health problems in Kenya. According to the World Health Organization (WHO), in 2002, there were over 124,000 reported cases of malaria nationwide in a population reported at just over 32 million [1]. Moreover, cases are likely grossly underreported [2]. According to one study, the mortality from malaria in one area of the Lake Victoria Basin was 20.8 per 1000 person-years [3]. The malaria burden is increased by high levels of resistance to antimalarial drugs [4-9] and by frequent self-administration of antimalarials [10-17].

Between June and September 2004, researchers from the Kenya Medical Research Institute (KEMRI) and the United States Army Medical Research Unit – Kenya (USAMRU-K) carried out a community-based malaria parasitaemia and arboviral serologic survey of residents of three ecologically and culturally diverse Kenyan districts: Samburu, Malindi, and Busia, to assess potential socioeconomic risk factors for exposure to these pathogens. Extensive demographic, socioeconomic, health history, and recent antimalarial use data were collected along with blood for malaria smear and arboviral antibody testing. The malaria parasitaemia rates found in these three districts are reported here. Additionally, this study examines individual risk factors for parasitaemia, such as occupation, literacy, recent illness, health care utilization, and use of antimalarials.

## Methods

### Study design

The study employed a population-based, cross-sectional design. Two villages were selected from each of the three districts under study. A two-stage cluster sampling technique was used to select villages for enrollment. The study population enrolled consisted of all consenting adults aged 18 years and above living in any of these six villages for at least five years. A standardized questionnaire was administered and a malaria smear taken at enrollment. This study was performed under a protocol approved by the KEMRI/National Ethical Review Committee.

### Site selection

#### District

The districts of Samburu, Malindi and Busia were selected for their ecological and social diversity. Samburu is located in semi-arid northern Kenya, where malaria is mesoendemic, and is sparsely populated, mostly by semi-nomadic herdsmen. Malindi is an Indian Ocean coastal district, with endemic malaria and a population of mostly fishermen and agriculturalists. Busia is located in the Lake Victoria basin region on the Ugandan border. It is a center of cross-border commerce and trade, but also has a large agrarian population and is endemic for malaria.

#### Village

Kenya is divided into provinces, districts, subdistricts, divisions, locations, sublocations and villages. To obtain a representative sample of adults residing in the districts, a two-stage cluster sampling technique was used. A sampling frame was created for each district, from which two sublocations were selected using probability proportional to population size sampling without replacement [18]. The second stage sampling frames were then created for each of these six sublocations with the enumeration units being the sublocation's villages. Using the same sampling technique, one village from each sublocation was selected. All eligible and consenting adults from each selected village were enrolled.

### Subject recruitment and informed consent

At each of the selected sites, both the community leaders and the community were engaged prior to conducting the study. These leaders organized community meetings at which study staff explained the study and answered any questions posed in this open forum.

Enrollment was conducted in community centers, such as schools or clinics, selected by local community leaders. These leaders verified that all potential subjects had been residents of the selected villages for at least five years. Before any potential subject was enrolled, a trained field worker explained the study in the local language and offered the opportunity for potential subjects to ask questions. Investigators were available to answer any questions posed. Witnessed informed consent was obtained if the potential subject demonstrated understanding of the study and was willing to enroll. In the case of an illiterate subject, a thumbprint was obtained on the consent forms and a separate Witness to Consent form was signed by a literate witness who had observed the consent process.

### Questionnaire

The study questionnaire was developed to obtain demographic, socioeconomic, and health information. Local medical authorities participated in questionnaire design. The questionnaire was modeled partly on the Kenya Demographic and Health surveys: 1988–1989 [19], 1998 [20], and 2002 [21]. The questionnaire was piloted in communities similar to those in the actual study, and was administered by trained interviewers. Before the interview ended, a principal investigator verified that recorded questionnaire responses were clear, complete, and internally consistent.

### Laboratory

Thick and thin smears were prepared and read on-site by the project's laboratory technicians. A smear was considered to be negative if no *P. falciparum *parasites were observed in 100 high-powered fields. Subjects with positive smears were treated, free of charge, with an artemisinin-containing combination antimalarial, as recommended by the Kenyan Ministry of Health. This treatment constituted the study's direct benefit to the participants.

### Data analysis

Questionnaire information included a categorical age variable (18–25, 26–35, 36–45, and 46+ years old), sex, literacy (defined as being able to read or write a simple line and being able to read a newspaper), and occupational group. Subjects reported whether or not they had been ill in the past three months and, if so, what illness they had (unsure, malaria, common cold, diarrhoea, HIV/AIDS, STI, respiratory, other). Additionally, subjects were asked to report whether they had used antimalarials in the past month and which antimalarials were taken. Malaria smear results were examined as a dichotomous variable (positive or negative).

Questionnaire and laboratory data were double-entered into a Microsoft Access^® ^(Microsoft Co., Redmond, WA) database and cross-checked for accuracy. Stata version 7 for Windows (Stata Corp., College Station, TX) was used for all statistical analysis. To obtain a valid estimate of associations between exposure variables and parasitaemia, univariate analysis was performed with Pearson's Chi-square test and univariate logistic regression. Mantel-Haenszel stratified relative odds (ROs) were calculated prior to further multivariate analysis to detect possible interaction between variables examined. As no interaction was detected, these results are not reported. A multivariate logistic regression model was then created which included only variables found to be at least marginally significant in univariate analysis (district, literacy, occupation, illness in previous three months), calculating maximum likelihood estimates of the adjusted ROs and 95% confidence intervals. If a categorical variable did not demonstrate a log-linear correlation with the outcome, dummy dichotomous variables were created and used in the logistic regression model. A p-value of <= 0.05 is considered statistically significant, while a p-value of > 0.05 and <= 0.10 is reported as marginally significant.

## Results

### Study population

A total of 1,141 subjects were enrolled, representing 27.8% of the adult populations of the selected villages: 238 from Samburu, 442 from Malindi, and 461 from Busia. Of these, 731 (64.1%) were female and 410 (35.9%) were male. There was a marked difference in sex distribution in the different age ranges (p < 0.001), with younger males being less proportionally represented than younger females. However, there was no statistically significant difference in the age or sex distribution of subjects among the three districts (p = 0.104, p = 0.909, respectively). Of the 814 who answered questions about literacy, 322 (39.6%) were literate. Of the 1,110 subjects who responded to occupational questions, 585 (52.7%) were involved in agriculture, followed by home duties, which accounted for 167 (15.1%). No other occupational groups constituted more than 10% of the study population, so these other groups are not analysed separately.

### Malaria smear results

Malaria smear results are summarized in Table 1.

**Table 1:** Parasitaemia rates and relative odds with 95% confidence intervals.

Characteristic	n	Parasitemia rate	Unadjusted RO	Adjusted RO*
District				
Samburu	238	1.7%	Reference	
Malindi	442	7.2%	4.57 (1.59 – 13.07)	4.03 (1.35 – 12.02)
Busia	461	22.3%	16.83 (6.12 – 46.32)	14.41 (5.04 – 41.21)
Age				
18 – 25	266	13.2%	Reference	
26 – 35	286	12.6%	0.95 (0.58 – 1.56)	
36 – 45	219	12.3%	0.93 (0.54 – 1.59)	
46+	370	11.1%	0.82 (0.51 – 1.33)	
Sex				
Female	731	11.5%	Reference	
Male	408	13.2%	1.17 (0.82 – 1.69)	
Literacy				
Illiterate	492	6.9%	Reference	
Literate	322	13.7%	2.13 (1.33 – 3.42)	1.78 (1.08 – 2.92)
Occupation				
Other	458	12.8%	Reference	
Agriculture	585	14.4%	1.15 (0.77 – 1.70)	
Home Duties	167	2.4%	0.17 (0.06 – 0.48)	0.76 (0.44 – 1.31)
Illness in Past 3 Months				
Other	789	10.5%	Reference	
Diarrhoea	45	25.9%	2.97 (1.22 – 7.24)	2.31 (0.94 – 5.68)
Malaria	307	15.3%	1.49 (1.00 – 2.20)	1.06 (0.61 – 1.84)
Antimalarial Use Past 4 Weeks				
Not Used	954	11.9%	Reference	
Used	187	13.3%	1.14 (0.71 – 1.81)	

* Modeled relative odds in logistic regression model.

#### District

Of the 1,141 subjects tested, 139 (12.2%) had a positive smear for *P falciparum*. The rate of positives varied markedly by district (p < 0.001), with 1.7% positive in Samburu, 7.2% in Malindi, and 22.3% in Busia.

#### Sociodemographics

Those who were literate had significantly higher parasitaemia rates than nonliterates with 13.7% of literates positive but only 6.9% of illiterates (p = 0.001). The rates of parasitaemia were lower in those engaged in home duties, at 2.4%, than those in agriculture, at 14.4%, or other employment areas, at 12.8% (p < 0.001). Neither age nor sex were associated with differences in parasitaemia rates (p = 0.872, p = 0.387, respectively).

#### Recent Illness History

Those who reported either a malarial or a diarrhoeal illness in the past three months had significantly increased rates of parasitaemia (p = 0.050, p = 0.010, respectively). Of the 307 with a history of malaria, 15.3% were parasite positive on enrollment, while for the 45 reporting diarrhoea, the parasitaemia rate was 25.9%. The parasitaemia rate for those with other reported illness or no illness was 10.5%.

#### Antimalarials

Among those who had taken antimalarials in the past four weeks and those who had not, 13.3% and 11.9%, respectively, were smear-positive (p = 0.625). Analysis for each medication separately failed to demonstrate any statistically significant association with smear positivity (p = 0.603).

### Logistic regression models

Relative odds and 95% confidence intervals are presented in Table 1. A multiple logistic regression model that included variables found to be significant in univariate analysis demonstrated a statistically significant increased relative odds of parasitaemia for subjects in Malindi (p = 0.012) and Busia (p < 0.001) and those who were literate (p = 0.023), and a marginally significantly increased odds of parasitaemia in those with diarrhoeal illness in the previous three months (p = 0.067). No association remained of parasitaemia with primary occupation of home duties (p = 0.327) or with history of malaria in the previous three months (p = 0.849).

## Discussion

Overall, this study found an association between parasitaemia and district of residence, literacy, and a history of diarrhoeal illness in the previous three months.

An interesting finding of this survey is that a history of diarrhoeal illness is a significant predictor for parasitaemia in Kenyan adults. The literature on the association of diarrhoea with malaria is somewhat conflicting and generally refers to the relationship between diarrhoea and acute malaria, while this survey correlates a history of diarrhoea in the past three months with a current parasitaemia. While diarrhoea is an often-cited symptom of malaria [22-24], there are several studies in the literature which demonstrate a negative correlation or lack of association between diarrhoea and malaria, both in non-immune travelers and in residents of endemic areas [25-28]. It is important to ascertain whether malaria is presenting as diarrhoeal illnesses in these populations to avoid missing the diagnosis of malaria and to avoid unnecessary antibacterial therapy for diarrhoea.

This positive relationship between a history of diarrhoea in the past three months and a positive malaria smear reached only marginal statistical significance in the aggregate, but was consistent in the individual districts, making type I error an unlikely explanation for this association. There are certainly many possible explanations for this association. It is of considerable interest that self-reported diarrhoea was associated with smear-positivity, whereas self-reported malaria was not. In these semi-immune populations, it is quite possible for diarrhoea to be the predominant symptom of malaria. As a diarrhoeal illnesses may not be recognized as malaria, treatment for malaria may not be sought or provided. While this difference in obtaining antimalarial therapy could explain a higher risk of parasitaemia among those with self-reported diarrhoea than self-reported malaria, this study does not support this as the explanation. While 30% of those with a history of malaria report taking an antimalarial in the previous month, 29% of those with a history of diarrhoea also report doing so. This difference is not statistically significant (p = 1.000). However, both of these groups were more likely to have taken an antimalarial than those with other recent reported illnesses, of whom 14% took antimalarials (RO = 2.62, 95% CI 1.84 – 3.75) or those with no reported illness, of whom 6% took antimalarials (RO = 6.52, 95% CI 4.06 – 10.48). Of those with a history of either diarrhoea or malaria, those with a positive malaria smear were no more or less likely to have taken an antimalarial in the past month than those with a negative smear (p = 0.531, p = 0.708). There was no difference in the antimalarials taken by those with diarrhoea and those with malaria (p = 0.869). Further study of this association of diarrhea with parasitaemia would be useful in providing guidance for evaluation of diarrhoea in these populations and perhaps would prevent inappropriate antibiotic therapies from being administered.

Literacy was another significant risk factor for parasitaemia in this study, even after controlling for potential confounding effects. This was a consistent finding in all three districts, although not reaching statistical significance for individual districts. This association cannot be attributed to occupation, recent antimalarial treatment, or other known factors. Literacy can directly impact health-seeking behaviors and also serves as a marker for other socioeconomic differences between individuals. It is plausible that differences in health-seeking behaviors or other socioeconomic factors would affect rates of malaria infection. However, literacy would have been anticipated to be protective against, not a risk factor for, infection as literacy and education are believed to be associated with more appropriate use of medical care [29-35]. There is some evidence, though, that higher educational level does not correlate with appropriate judgment of illness severity [36]. This observation of higher parasitemia rates among literate subjects warrants further study.

A clear finding of this study is that district of residence is the factor most strongly associated with differences in parasitaemia rates at the time of enrollment. Specifically, compared to those enrolled in Samburu, the subjects enrolled from Busia and, less so, Malindi, had higher odds of parasitaemia, even after adjustment for known potential confounders. The difference in ecologies of a semi-arid, a coastal, and a Lake Victoria basin district could well result in different disease transmission rates, particularly for vector-borne diseases [37-39]. District differences in public health infrastructure, sanitation, and cultural practices could also account for differences in disease occurrence and health perceptions.

There was no statistically significant association between history of antimalarial use in the past four weeks and the presence of parasitaemia on enrollment. This study did not have sufficient power to detect an association between specific antimalarial use and parasitaemia, even if such an association actually existed. Given an overall rate of 12.2% positivity, to find a 5% difference in malaria positivity with a power of 80% would require 826 subjects with a history of antimalarial use, a much higher number than the 187 enrolled.

There are several further limitations of this study. First is the use of self-reported data, particularly in the area of recent health histories and antimalarial usage. As noted above, the low number of patients with a recent history of antimalarial use limits the study's ability to investigate antimalarial resistance. Also, as previously noted, the enumeration unit was the village, not the individual subject.

## Conclusion

While district of residence was the variable most highly predictive of parasitaemia among Kenyan adults surveyed, both a recent history of diarrhoeal illness and literacy were at least marginally statistically significant predictors. No other demographic or occupational variable, including age and sex, was associated with parasitaemia in these populations. Further study is required to determine the nature of these associations.

## Authors' contributions

RLC served as overall Principal Investigator on the study, involved in all aspects of protocol design and execution, data analysis, and manuscript preparation. TP and FO designed the sociodemographic questionnaire, supervised field workers, and assisted in manuscript preparation. VOO designed the laboratory system and supervised the reading of malaria slides. NA conceived the study, supervised field and data entry personnel, and provided significant contribution to the manuscript. All authors read and approved the final manuscript.
